# Early childcare arrangements and children's internalizing and externalizing symptoms: an individual participant data meta-analysis of six prospective birth cohorts in Europe

**DOI:** 10.1016/j.lanepe.2024.101036

**Published:** 2024-08-21

**Authors:** Katharine M. Barry, Demetris Avraam, Tim Cadman, Ahmed Elhakeem, Hanan El Marroun, Pauline W. Jansen, Anne-Marie Nybo-Andersen, Katrine Strandberg-Larsen, Llúcia González Safont, Raquel Soler-Blasco, Florencia Barreto-Zarza, Jordi Julvez, Martine Vrijheid, Barbara Heude, Marie-Aline Charles, Alexandre Ramchandar Gomajee, Maria Melchior

**Affiliations:** a Sorbonne Université, Paris, France; b French National Institute of Health and Medical Research (INSERM), Institut Pierre Louis d'Epidémiologie et de Santé Publique (IPLESP), Equipe de Recherche en Epidémiologie Sociale (ERES), Paris, France; c Section of Epidemiology, Department of Public Health, University of Copenhagen, Copenhagen, Denmark; d Integrative Epidemiology Unit at the University of Bristol, Bristol, UK; e Department of Child & Adolescent Psychiatry/Psychology, Erasmus Medical Center (MC), University Medical Center Rotterdam, Rotterdam, the Netherlands; f Department of Psychology, Education and Child Studies, Erasmus University Rotterdam, Rotterdam, the Netherlands; g Nursing and Chiropody Faculty of Valencia University, Valencia, Spain; h Joint Research Unit in Epidemiology, Environment and Health (FISABIO-UJI-UV), Valencia, Spain; i Valencia University, Valencia, Spain; j Biomedical Research Centre Network for Epidemiology and Public Health (CIBERESP), Madrid, Spain; k Faculty of Psychology, University of the Basque Country (UPV/EHU), San Sebastian, Spain; l Institute for Global Health (ISGlobal), Barcelona, Spain; m UMR1153 Center for Research in Epidemiology and Statistics (CRESS), Paris, France; n Early Life Research on Later Health Team (EARoH), Paris, France; o Joint ELFE Unit (INSERM), French National Institute for Demographic Studies (INED), Aubervilliers, France; p French Blood Establishment (EFS), Aubervilliers, France; q French School of Public Health (EHESP), Doctoral Network, Rennes, France; r Spanish Consortium for Research on Epidemiology and Public Health (CIBERESP), Spain; s Epidemiology and Environmental Health Joint Research Unit (FISABIO-Universitat Jaume I-Universitat de Valencia), Valencia, Spain; t Environmental Epidemiology and Child Development Group, Biodonostia Health Research Institute, San Sebastian, Spain; u Pere Virgili Institute for Health Research (IISPV), Universitat Rovira i Virgili, Tarragona, Spain

**Keywords:** Centre-based childcare, Informal childcare, Internalizing symptoms, Externalizing symptoms, Epidemiology, Child cohorts

## Abstract

**Background:**

Early childcare attendance may be related to children's internalizing and externalizing symptoms throughout childhood and young adolescence, however evidence from Europe is limited. We aimed to assess this association across multiple population-based birth cohorts of children recruited in different European countries.

**Methods:**

Data come from six parent-offspring prospective birth cohort studies across five European countries within the EU Child Cohort Network. A total of 87,208 parent-child dyads were included in the study. To test associations between childcare attendance (centre-based or informal) anytime between ages 0 and 4 years and children's internalizing and externalizing symptoms in middle childhood and young adolescence (measured at: 5–6 years, 7–9 years, and 10–13 years) a two-stage individual participant data meta-analysis was implemented. Linear regression models were performed in each cohort separately; combined random-effects meta-analysis was then used to obtain overall association estimates. In secondary analyses, we tested interactions between childcare attendance and mother's post-partum depression, low education status, and the child's sex.

**Findings:**

Compared to children who were exclusively cared for by their parents prior to school entry, those who attended centre-based childcare had lower levels of internalizing symptoms in all age groups [5–6 years: β: −1.78 (95% CI: −3.39, −0.16); 7–9 years: β: −0.55 (95% CI: −0.88, −0.73); 10–13 years: β: −0.76 (95% CI: −1.15, −0.37)]. Children who attended informal childcare appeared to have elevated levels of internalizing symptoms between 7–9 and 10–13 years, respectively [β: 1.65 (95% CI: 1.25, 2.06); β: 1.25 (95% CI: 0.96, 1.54)]. Informal childcare attendance was also associated with increased levels of children's externalizing symptoms between 7–9 and 10–13 years, respectively [β: 2.84 (95% CI: 1.41, 4.26); β: 2.19 (95% CI: 0.54, 3.84)].

**Interpretation:**

Early centre-based childcare is associated with decreased levels of children's internalizing symptoms compared to exclusive parental care. For informal childcare, opposite associations were observed. Overall, our results suggest that centre-based childcare attendance may be associated with slight positive impacts on children's emotional development and should be encouraged by public policies. In addition, children from socioeconomically disadvantaged families require special attention, as they may not sufficiently benefit from universal early childhood education and care (ECEC).

**Funding:**

This research was funded by the ERC Consolidator grant RESEDA (Horizon Europe, 101001420).

**Research in context**

**Evidence before this study**

High quality, early non-parental childcare has been associated with children's improved cognitive and language abilities as well as positive peer interactions. However, most of the evidence comes from the United States and Canada and may not be broadly generalizable. Past studies vary in terms of definitions and timing of follow-up of children's psychological development. We searched PUBMED and Google scholar on April 28th, 2024 for articles related to early childcare and children's internalizing and externalizing behaviors across childhood using the following search terms: ((((((((early childcare) OR (nonparental childcare)) OR (early childcare arrangement)) AND (internalizing)) OR (externalizing)) OR (internalising)) OR (externalising)) OR (behavior)) OR (emotional development). We found limited evidence from Australia, North America, Norway, and Switzerland, with only one longitudinal study from the United States. Some studies showed that high quality early childcare is linked to positive prosocial skills and lower levels of internalizing symptoms, while others found no relationship. Additionally, most studies did not differentiate between centre-based and other types of childcare, making it difficult to determine the impact of structured, professional settings on children's socio-emotional development.

**Added value of this study**

Using longitudinal data from six birth cohorts implemented in Britain, the Netherlands, Denmark, France, and Spain, we examined the impact of attendance of centre-based or informal childcare on children's internalizing and externalizing symptoms from middle childhood to early adolescence (5–6 years, 7–9 years, 10–13 years). Children who attended early centre-based childcare between ages 0 and 4 years had lower levels of internalizing symptoms later on compared to those in exclusive parental care. Conversely, informal childcare attendance was associated with higher levels of internalizing and externalizing symptoms between ages 7–9 and 10–13 years. Internalizing and externalizing symptoms levels were higher among children whose mother had low educational levels and lower among girls. This study adds valuable information for Europe, as most research on early childcare and children's development. The six countries included in this study have national standards for center-based childcare, ensuring more homogenous definitions and quality of care than in previous investigations conducted in North America.

**Implications of all the available evidence**

To our knowledge, ours is the first individual participant meta-analysis to test the prospective relationship between early childcare arrangements and children's internalizing and externalizing symptoms in five European countries. Our results suggest that early centre-based childcare attendance may protect children from later internalizing difficulties. However, childcare attendance does not appear to compensate socioeconomic inequalities with regard to psychological development which appear early in life.

## Introduction

In the European Union (EU) in 2020, approximately 53.4% of children under the age of three years were exclusively taken care of by their parents, 32.3% were in formal childcare for at least 1 h per week and 20.9% were cared for by grandparents, other relatives, or professional childminders.^1^ Childcare, defined as any care a child receives outside of his/her parents, is often divided into institutionalized childcare (that is centre-based) or informal childcare (a childcare professional, relatives/friends/nanny/babysitter/au pair and/or someone other than the parents). Understanding how non-parental childcare impacts a child's development may provide families and policymakers with valuable information on whether increasing availability of non-parental childcare can benefit not only parents' work-life balance but also children's socio-emotional and cognitive development.

High quality childcare attendance–particularly centre-based–has been associated with children's improved cognitive and language acquisition skills and later school achievement,^2^, ^3^, ^4^, ^5^ as well as fewer peer problems and lower levels of emotional difficulties.^6^^,^^7^ Centre-based childcare can provide children with an array of learning opportunities that they may not have at home or with a nanny^5^^,^^8^ and especially benefit those who come from disadvantaged backgrounds and experience family adversity.

The long-term impact of early childcare arrangements on children's emotional development is of interest due to the possible negative repercussions of early onset internalizing and externalizing symptoms. Internalizing symptoms can include signs of anxiety/depression, withdrawn behavior and/or somatic complaints.^9^ Externalizing symptoms can include attention difficulties and delinquent and/or aggressive behaviors.^9^ One study found a prevalence of 18.4% of internalizing and 7.8% of externalizing symptoms among children aged 6–12 years in eight EU countries.^10^ Children who experience internalizing symptoms in early adolescence are at higher risk of alcohol dependence, lower intimate partnership, higher welfare benefits and psychopathology later in life.^11^^,^^12^ Children who display externalizing symptoms from early on are at risk of decreased opportunities for social growth, lower school achievement, worse psychosocial outcomes, and less financial stability in their 20s and early 30s.^11^ Considering the high prevalence of internalizing and externalizing symptoms in children and the possible detrimental health and social consequences they can have, there is need to identify propitious population-based protective factors, possibly childcare attendance prior to school entry, that could protect against internalizing and externalizing symptoms. Additionally, it is important to explore whether maternal postpartum depression, maternal educational level, and child's sex modify the relationship between early childcare arrangements and children's internalizing and externalizing symptoms. Children with depressed mothers are at higher risk for poor cognitive development, internalizing symptoms, difficult temperaments, and poorer language development, and some studies have suggested that early childcare arrangements may be able to buffer these effects.^13^, ^14^, ^15^ Similarly, maternal education level can also be explored as a moderator because disadvantaged backgrounds often lead to worse socio-emotional and cognitive outcomes and studies have yielded mixed results on whether children benefit more or less from childcare depending on their family's socio-economic background.^4^^,^^5^^,^^8^ Lastly, differences between boys and girls in internalizing and externalizing symptoms call for examining if the child's sex modifies these relationships.^16^^,^^17^

Our aim was to examine the relationship between early childcare attendance prior to school entry and children's internalizing and externalizing symptoms in middle childhood and early adolescence, testing the hypothesis of a protective effect of centre-based childcare in a European context. We used data from five EU countries to test: (i) whether centre-based childcare and informal childcare are associated with lower levels of internalizing and externalizing symptoms compared to exclusive parental childcare, and (ii) whether mother's post-partum depression, educational level, or the child's sex modify the association between early childcare attendance and children's internalizing and externalizing symptoms.

## Methods

### Study design and oversight

**Fig. 1:**
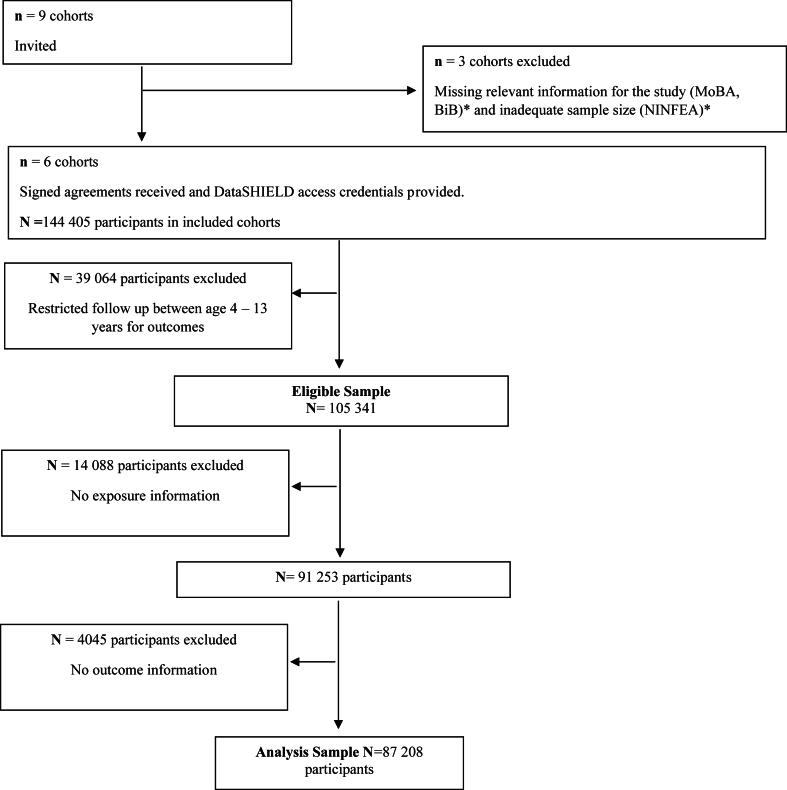
Flow chart of cohorts and participants from the EU Child Cohort Network included in the study of the relationship between access to childcare and children’s internalizing and externalizing symptoms. ∗MoBA, Norwegian Mother, Father, and Child Cohort Study; BiB, Born in Bradford Family Cohort; NINFEA, Nascita e Infanzia: gli Effetti dell'Ambiente" (Birth and Childhood: the Effects of the Environment) Cohort Study.

This study includes six parent-offspring prospective birth cohort studies across five European countries participating in the EU Child Cohort Network (EUCCN).^18^ The EUCCN is part of the EU Horizon 2020 LifeCycle project, funded in 2017 with the overarching aim of understanding relationships between early-life stressors and child development, health and disease throughout the life course. Parent-offspring cohort studies were included in the study if they (i) collected information about non-parental childcare before school entry and recorded children's internalizing and externalizing symptoms at least once during follow-up, (ii) were approved by their institutional review boards, and (iii) had harmonized data available for analysis via the federated data analysis platform DataSHIELD^19^ (Fig. 1). Participating cohorts include: the Avon Longitudinal Study of Parents and Children^20^ (ALSPAC; recruitment from 1 April 1991 to 31 December 1992) in the United Kingdom, The Generation R study^21^ (GENR; recruitment from 1 April 2002 to 31 January 2006) in the Netherlands, the Danish National Birth Cohort^22^ (DNBC; recruitment from 1995 to 2002) in Denmark, the INfancia y Medio Ambiente Project^23^ (INMA; recruitment from 1997 to 2008) in Spain, the Étude des Déterminants pré Et postnatals du Développement de la santé de l'enfant^24^ (EDEN; recruitment from 27 January 2003 to 6 March 2006) in France, and the Étude Longitudinale Française depuis l'Enfance^25^ (ELFE; recruitment on 25 selected days of 2011 spread over the year) in France. All participating cohorts received approval from their local institutional review boards, written informed consent from all participants and parents was obtained, and written informed consent for use of data in this project was obtained from all principal investigators. Information regarding the ethical approval and consent from all participants has been outlined in the Supplementary Information (pages 1–3). The study design and characteristics of participants taking part in each cohort have been described in detail elsewhere.^20^, ^21^, ^22^, ^23^, ^24^, ^25^

### Participants

After multiple imputation of missing data on study covariates (Supplementary Table S1), a total of 87,208 participants from six-participating parent-offspring cohorts were used to study internalizing and externalizing symptoms (Fig. 1).

### Measures

#### Exposures of interest

##### Non-parental childcare care attendance

Parents reported on their children's childcare arrangements at least one time between the ages of 0–4 years (Supplementary Table S2). If the parent reported that their child was in centre-based childcare, he/she was included in the “centre-based childcare” group [yes/no]. If the parent reported that their child was in informal rather than in centre-based childcare, including, a childcare professional, relatives/friends/nanny/babysitter/au pair and/or someone other than the parents, they were considered to be in “informal childcare” [yes/no]. Supplementary Table S3 provides some contextual information on each cohort's country's early childcare policies at the time of data collection.

#### Outcomes of interest

##### Children's internalizing and externalizing symptoms

Children's internalizing and externalizing symptoms were reported by parents using either the Strengths and Difficulties Questionnaire (SDQ)^26^ or the Child Behavioral Checklist (CBCL).^27^ Information regarding the use of different measures, ages of assessment and the standardization process for the scores is shown in Supplementary Table S2.

##### Covariates

Covariates were selected a priori based on previous research findings: maternal age in years was collected at the child's birth. Other maternal measurements were assessed in the first 12 months of the child's life: educational level: *high (associate, bachelor, masters, doctoral or equivalent), intermediate (upper secondary, post-secondary non-tertiary) or low (no education, early childhood, pre-primary, primary, lower secondary or second stage of basic education)*, based on the International Standard Classification of Education 97/2011 (ISCED-97/2011),^28^^,^^29^ employment status: *[employed, unemployed, other (student, apprentice, domestic tasks, inactive/receiving benefits, etc.)]*, parental separation: *did the parents split up during the first year of the child's life [yes/no])*, maternal post-partum depression (PPD) within the first year after child's birth [yes/no], and whether the participating child was an only child [yes/no].

The child's sex [male/female], birth weight (in grams), and gestational age (in weeks) were recorded at the child's birth.

### Statistical analyses

To study the relationship between early childcare arrangements and children's internalizing and externalizing symptoms, we implemented a two-stage individual participant data (IPD) meta-analysis in order to reduce the risk of trial-level confounding being introduced into the analysis.^30^

To address missing information on covariates (Supplementary Table S1), ten imputed datasets using fully conditional specification to impute missing data on covariates were performed for each sample. The imputation model incorporated all covariates and outcomes included in the analytical models. Second, linear regression models were implemented in each cohort separately. Third, cohort-specific coefficients and standard errors were combined using random-effects meta-analysis with a restricted estimate maximum likelihood (REML) approach to attain overall effect estimates. Based on the timing of measurement of children's internalizing and externalizing symptoms in each cohort, we distinguished three age-groups (5–6 years, 7–9 years, 10–13 years). In the ALSPAC, GENR, EDEN, DNBC and ELFE cohorts, regression models were adjusted for maternal age, educational level, employment status, parental separation, maternal PPD, only child status, child's birthweight, gestational age, and sex. In the INMA cohort, regression models were adjusted for previous variables except parental separation status, which was not measured.

The combined estimates testing associations between early childcare arrangements and children's internalizing and externalizing symptoms are presented with their accompanying 95% confidence intervals (95% CI), the estimated p-value and their heterogeneity statistics (Q, degrees of freedom (df), p-value, I^2^, H^2^). An I^2^ statistic >75% or I^2^ ∼ 75% with effect estimates in different directions represents moderate to substantial heterogeneity.^31^^,^^32^

In sensitivity analyses, we tested association between childcare type from 0 to 3 years and children's internalizing and externalizing symptoms between 5–6 years, 7–9 years, and 10–13 years, as some countries, such as France, Spain, and the Netherlands, have different standards, structures, and regulations for childcare from 0 to the age of 3 years.^33^ Additionally, we examined whether associations between childcare attendance and children's internalizing and externalizing symptoms were modified by maternal PPD, low education status (classified as any education at or below upper secondary, post-secondary or non-tertiary school) (yes/no) or the child's sex by including an interaction term (early childcare type∗interaction of interest) in separate models for each interaction.^34^^,^^35^ Lastly, a sensitivity analysis was carried out in which cohorts were stratified based on the type of assessment tool was used to measure internalizing and externalizing symptoms to see if there were significant differences from our main results. The DNBC, ELFE, and EDEN cohorts used the SDQ assessment tool, while the ALSPAC and GENR cohorts used the CBCL assessment tool for all age groups. The INMA cohort used the SDQ assessment tool for ages 7–9 years and the CBCL assessment tool for ages 10–12 years.

All statistical analyses were performed using DataSHIELD (packages: dsBase version 6.3.0; dsHelper version 1.4), a tool for federated analyses of individual-level data.^19^^,^^36^ All statistical codes used to analyze data for this project have been made publicly available: https://github.com/katybarry/nonparental-care-attendance-and-childrens-behaviors.git.

### Role of the funding source

The funders had no role in the design and conduct of the study; the analysis and interpretation of the data; preparation; the review, or approval of the manuscript.

## Results

### Participants’ characteristics

We analyzed a total of 87,208 mother-child dyads, with 24,651 reporting information on children's internalizing and externalizing symptoms between 5 and 6 years, 65,081 between 7 and 9 years, and 50,896 between 10 and 13 years. Table 1 shows combined sample characteristics of participants by age group.

**Table 1:** Covariate, exposure and outcome information in the EU Child Cohort Network (ALSPAC, GENR, DNBC, INMA, EDEN, ELFE)^a^ in the study of the association between childcare attendance and children's emotional and behavioural difficulties (n, %).

Age group	Combined study sample, N = 87,208		
	5–6 years (n = 24,651)	7–9 years (n = 65,081)	10–13 years (n = 50,896)
Included cohorts	4	5	4
Mother's age, median, (IQR)	30.63 (33.77, 26.57)	30.04 (33.04, 27.11)	29.90 (32.89, 26.91)
Mother's educational level			
High, n (%)	12,317 (50.00)	29,476 (45.30)	23,624 (46.40)
Medium, n (%)	10,044 (40.70)	26,676 (41.00)	20,729 (40.70)
Low, n (%)	2290 (9.29)	8929 (13.70)	6543 (12.90)
Mother's employment status			
Employed, n (%)	18,816 (76.32)	49,412 (75.92)	38471 (75.59)
Unemployed, n (%)	795 (3.22)	2972 (4.57)	2139 (4.20)
Other, n (%)	5040 (20.44)	12,697 (19.51)	10,286 (20.21)
Mother post-partum depression (yes)	2022 (8.20)	9017 (13.90)	7159 (14.10)
Parental separation (yes), n (%)	472 (1.91)	839 (1.31)	604 (1.20)
Child's sex (female), n (%)	12,073 (49.00)	31,814 (48.90)	25,556 (50.20)
Only child (yes), n (%)	11,971 (48.60)	31,447 (48.30)	24,421 (48.00)
child's gestational week of birth (in weeks)	40 (41,39)	40 (41, 39)	40 (41, 39)
Child's birthweight in grams, median (IQR)	3392 (3713,3076)	3563 (3905, 3240)	3571 (3915, 3251)
Types of childcare			
Centre-based childcare, n (%)	7194 (29.20)	17,137 (26.30)	12,594 (24.70)
Informal childcare, n (%)	13,008 (52.80)	33,006 (50.70)	26,360 (51.80)
Exclusive parental childcare, n (%)	4449 (18.00)	14,938 (23.00)	11,942 (23.50)
Internalizing symptoms percentile score, median (IQR)	46.82 (73.74, 19.30)	46.06 (66.19, 5.56)	43.55 (71.51, 23.87)
Externalizing symptoms percentile score, median (IQR)	48.51 (71.68, 20.75)	43.42 (70.75, 15.55)	39.84 (74.93, 20.59)

ALSPAC, Avon Longitudinal Study of Parents and Children; GENR, The Generation R study; DNBC, Danish National Birth Cohort; INMA, INfancia y Medio Ambiente Project; EDEN, Étude des Déterminants pré Et postnatals du Développement de la santé de l'enfant; ELFE, Étude Longitudinale Française depuis l'Enfance.

a Cohorts included in the combined sample.

### Children's internalizing symptoms

**Fig. 2:**
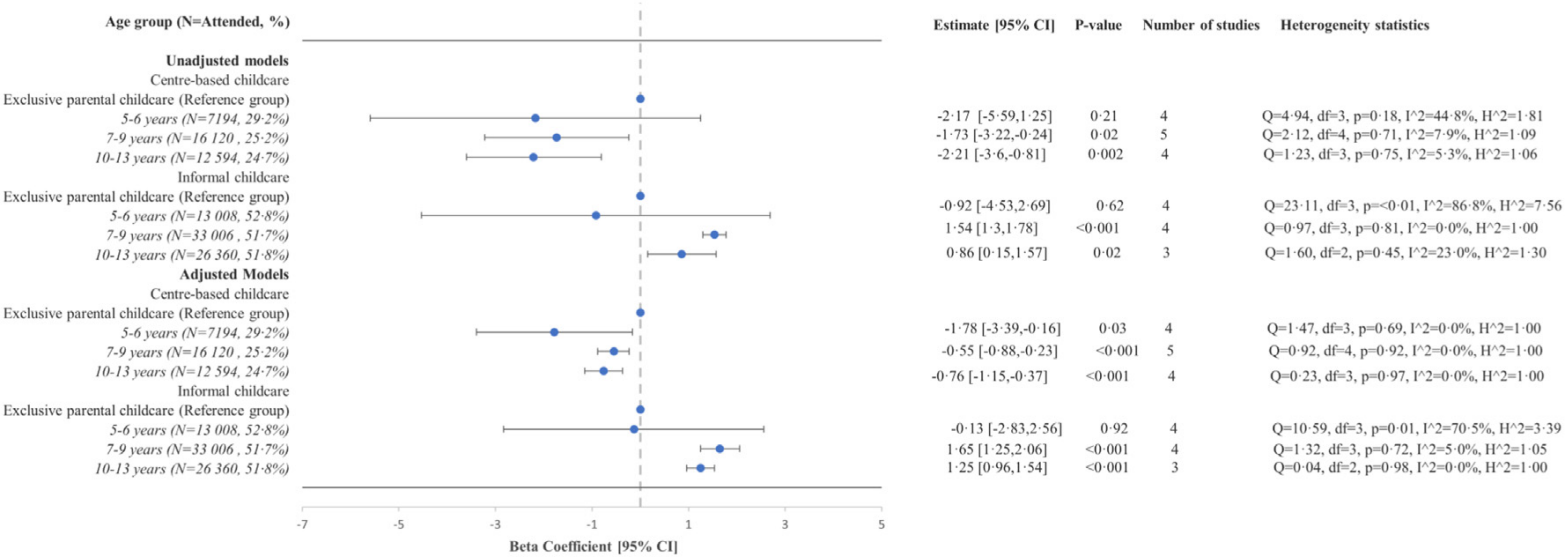
Crude and adjusted associations between childcare attendance between ages 0 up and age 4 and children’s internalizing symptoms by age group (5–6 years, 7–9 years, 10–13 years), in child cohort studies included in the EU Child Cohort Network (ALSPAC, GENR, DNBC, INMA, EDEN, ELFE)∗. ∗ALSPAC, Avon Longitudinal Study of Parents and Children; GENR, The Generation R study; DNBC, Danish National Birth Cohort; INMA, INfancia y Medio Ambiente Project; EDEN, Étude des Déterminants pré Et postnatals du Développement de la santé de l'enfant; ELFE, Étude Longitudinale Française depuis l'Enfance. A two-stage individual participant data (IPD) meta-analysis was performed on each age group presented. The reference group was children who were exclusively cared for by their parents. Linear regression models were performed separately on each cohort for each given age group and then cohort-specific coefficients and standard errors were combined using random-effects meta-analysis with a restricted estimate maximum likelihood (REML) approach to attain overall effect estimates. The beta coefficient is provided with the accompanying 95% Confidence Interval (CI) and p-value. The number of studies for each age bracket is provided as well as the heterogeneity statistics. For the adjusted models, the ALSPAC, GENR, EDEN, DNBC, and ELFE cohorts’ linear regression models were adjusted for maternal age at child’s birth, maternal education at child’s birth, maternal employment status at child’s birth, parental separation status, mother’s first child, child’s sex and child’s weight at birth when available. For INMA, models were adjusted for all the previous variables except for parental separation due to unavailability.

Compared to children who were cared for by their parents prior to age 4 years, those who attended centre-based childcare had lower levels of internalizing symptoms in all age groups: [5–6 years: β: −1.78 (95% CI: −3.39, −0.16); 7–9 years: β: −0.55 (95% CI: −0.88, −0.73); 10–13 years: β: −0.76 (95% CI: −1.15, −0.37)] (Fig. 2).

To the contrary, informal childcare attendance was associated with higher levels of internalizing symptoms between 7–9 years and 10–13 years, respectively: β: 1.65 (95% CI: 1.25, 2.06); β: 1.25 (95% CI: 0.96, 1.54) (Fig. 2).

### Children's externalizing symptoms

**Fig. 3:**
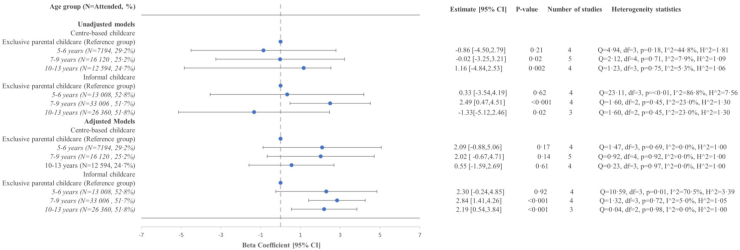
Crude and adjusted associations between anytime centre-based childcare and Informal childcare attendance between ages 0 up and age 4 and externalizing symptoms by age group (5–6 years, 7–9 years, 10–13 years), in child cohort studies included in the EU Child Cohort Network (ALSPAC, GENR, DNBC, INMA, EDEN, ELFE)∗. ∗ALSPAC, Avon Longitudinal Study of Parents and Children; GENR, The Generation R study; DNBC, Danish National Birth Cohort; INMA, INfancia y Medio Ambiente Project; EDEN, Étude des Déterminants pré Et postnatals du Développement de la santé de l'enfant; ELFE, Étude Longitudinale Française depuis l'Enfance. A two-stage individual participant data (IPD) meta-analysis was performed on each age bracket presented. The reference group was children who were exclusively cared for by their parents. Linear regression models were performed separately on each cohort for each given age bracket and then cohort-specific coefficients and standard errors were combined using random-effects meta-analysis with a restricted estimate maximum likelihood (REML) approach to attain overall effect estimates. The beta coefficient is provided with the accompanying 95% Confidence Interval (CI) and p-value. The number of studies for each age group is provided as well as the heterogeneity statistics. For the adjusted models, the ALSPAC, GENR, EDEN, DNBC, and ELFE cohorts’ linear regression models were adjusted for maternal age at child’s birth, maternal education at child’s birth, maternal employment status at child’s birth, parental separation status, mother’s first child, child’s sex and child’s weight at birth when available. For INMA, models were adjusted for all the previous variables except for parental separation status due to unavailability.

No associations were found between centre-based childcare and externalizing symptoms across all age groups (Fig. 3). Informal childcare attendance was associated with higher externalizing symptoms between 7–9 and 10–13 years, respectively: [β: 2.84 (95% CI: 1.41, 4.26); β: 2.19 (95% CI: 0.54, 3.84)] (Fig. 3). We observed potential heterogeneity in the unadjusted statistical models for the 5–6 (I^2^ = 90.2%) and 7–9 (I^2^ = 83.8%) years age groups which decreased after controlling for covariates [5–6 years (I^2^ = 72.1%) and 7–9 years (I^2^ = 67.7%)] (Fig. 3).

All cohort-specific associations are shown in the Supplementary Information (Supplementary Figures S1–S4).

### Early childcare from 0 to 3 years old

Studying specifically childcare attendance between the ages of 0 and 3 years, we found results similar to our main analysis. Children who attended centre-based childcare had lower levels of internalizing symptoms in all age groups: [5–6 years: β: −0.78 (95% CI: −1.15, −0.37); 7–9 years: β: −0.55 (95% CI: −0.87, −0.23); 10–13 years: β: −1.86 (95% CI: −3.42, −0.31)] (Supplementary Figure S5). To the contrary, informal childcare attendance was associated with higher levels of internalizing symptoms between 7–9 years and 10–13 years, respectively: β: 1.69 (95% CI: 1.45, 1.94); β: 1.25 (95% CI: 0.95, 1.54) as well as higher levels of externalizing symptoms at 7–9 and 10–13 years, respectively: [β: 2.77 (95% CI: 1.43, 4.11); β: 2.21 (95% CI: 0.52, 3.90)] (Supplementary Figure S5).

### Effect modification

We tested interactions between childcare attendance and mother's PPD, low education status (defined as having an intermediate education or below), and the child's sex (Supplementary Table S4). Regarding centre-based childcare attendance, we found a significant interaction between mother's low education status (yes) and centre-based childcare (p ≤ 0.01) with a positive beta coefficient (β = 1.27), indicating that children whose mother had a low educational level may have elevated levels of internalizing symptoms between 5 and 6 years. Regarding informal childcare attendance, we found statistically significant interactions with mother's low education status (yes) such that children whose mother had a low educational level were most likely to experience elevated levels of internalizing symptoms (5–6 years; 7–9 years; 10–13 years: p ≤ 0.001) with positive beta coefficients (5–6 years: β = 49.74, 7–9 years: β = 44.22, 10–13 years: β = 44.86). For externalizing symptoms at all age groups, we found significant interactions between mother's low education status (yes) and centre-based childcare (5–6: p = 0.03, 10–13: p = 0.01) with positive beta coefficients (5–6 years: β = 2.28, 10–13 years: β = 2.22). For externalizing symptoms at all age groups, we found statistically significant interactions between mother's low education status (yes) and informal childcare (5–6 years; 7–9 years; 10–13 years: p ≤ 0.001) with positive beta coefficients (5–6 years: β = 49.73, 7–9: β = 47.34, 10–13 years: β = 46.62). Lastly, the association between informal care attendance and children's externalizing symptoms was modified by child sex (10–13 years: p = 0.03, β = −1.21), such that girls were associated with lower externalizing symptoms if they attended informal childcare than boys.

### Stratification by outcome measurement tool

After stratifying the results based on the assessment tool used to measure children's internalizing and externalizing symptoms, we did not see substantial differences between the stratified groups and the main analysis as confidence intervals overlapped (Supplementary Table S5). We did, however, find that certain estimates became statistically significant after stratification. For example, cohorts which used the SDQ between ages 5 and 6 years (ELFE, EDEN) found that children who attended informal childcare had slightly decreased levels of internalizing symptoms [β: −2.12 (95% CI: −3.85, −0.38)]. Also, when cohorts were stratified by SDQ or CBCL, children who attended centre-based childcare had slightly higher externalizing symptoms between ages 7 and 9 years [β: 1.04 (95% CI: 0.75, 1.33)]; [β: 4.09 (95% CI: 0.53, 7.66)], respectively.

## Discussion

Studying six European mother-child cohorts which included 87,208 participants, we found that centre-based childcare attendance was associated with small decreases of internalizing symptoms; however, we observed the contrary regarding informal childcare attendance. Moreover, attendance at informal childcare was also associated with a small elevation of children's externalizing symptoms. It is important to note that in our study informal childcare, which is likely to vary across European countries, included occasional or regular care provided by relatives or friends, as well as care provided by nannies and childminders, implying that this group is characterized by heterogeneity. Unfortunately, we were not able to distinguish different subtypes of informal childcare as not all of the studies provided had this information. However, in the future it would be relevant to distinguish professional from non-professional types of informal childcare. Lastly, these results reflect childcare arrangements from the late 1990s and early 2000s. Therefore, it's crucial to consider the childcare context described in Supplementary Table S3 before generalizing these findings to the current childcare systems in each country.

The effects of centre-based and informal childcare appeared slightly stronger among children whose mothers had a low education level. Moreover, boys had slightly elevated externalizing symptoms between ages 10 and 13 years if they attended informal childcare compared to girls. Overall, these results suggest that centre-based childcare attendance is associated with small positive effects on children's later psychological well-being, but other unmeasured factors such as parenting styles and children's early temperament may also play a role in children's internalizing and externalizing symptoms.

Past scientific literature on relationships between attendance at early childcare arrangements and children's development has shown somewhat inconsistent findings depending on the type of childcare, the childcare setting and children's age.

Studies examining associations between centre-based childcare and children's internalizing symptoms in Europe reported protective effects and higher levels of prosocial skills.^6^^,^^34^^,^^35^^,^^37^^,^^38^ Children attending centre-based childcare may be more likely to learn emotional regulation, cooperation and conflict resolution skills compared to children attending informal childcare.^39^ Children in centre-based childcare may have access to better resources and facilities designed for children compared to informal child care which may home-based, and children in centre-based childcare may receive higher quality care due to the educational requirements and child-specific training that professional staff are required to have in centre-based childcare.^33^ The staff-to-child ratio in centre-based childcare may be higher than in other informal childcare settings meaning that children may receive more focused attention that could impact their socio-emotional development than in informal childcare.^33^ When considering socio-economic status, one study found that children who came from a more disadvantaged background experienced higher internalizing symptoms and substance use in childhood if they attended early centre-based childcare.^35^ The study explains that children from disadvantaged backgrounds may not experience the same benefits from childcare as their middle-class peers because childcare settings may mirror more middle-class behavioral norms, and as a result, these children may feel a sense of alienation as well as inner conflict as the behaviors and practices they are confronted with in childcare do not reflect the behaviors and practices they find in their home.^35^

Studying children's externalizing symptoms, a meta-analysis reported no association between the number of hours spent in centre-based childcare and children's externalizing symptoms below five years of age.^35^ In contrast, time spent in centre-based childcare has been reported to be related to higher levels of self-reported aggression between ages 7 and 8 years as well as self-reported Attention-Deficit/Hyperactivity Disorder (ADHD) symptoms between ages 15 and 17 years in Switzerland^35^ and higher levels of teacher-reported externalizing symptoms from 52 months to grade five in the United States.^38^

Some of the variation across studies may reflect differences in the quality, governance, accessibility and affordability of centre-based childcare provided for children before they start school in each country. Yet, only one-third of countries part of the Organization for Economic Co-operation and Development (OECD) have strict guidelines regarding the educational curriculum implemented in centre-based childcare centres as well as the developmental and learning goals that the childcare has set out to meet which are age-appropriate.^33^ Some countries, such as Denmark, France, and Spain, have curriculum themes that are implemented in childcare which include personal development, social development, communication and language, body, senses, and movement, nature, outdoor life and science, and culture, aesthetics and community.^40^, ^41^, ^42^ Other countries, such as the Netherlands, may not have any prescribed national curriculum, but may require childcare professionals to establish a curriculum plan that incorporates at least one child development goal.^43^ These curriculum themes and childcare activities may influence children's internalizing and externalizing symptoms later on through social development and emotional regulation skills acquired early on in life.^44^

Quality childcare as well as the number of children per centre-based childcare professional have been associated with fewer behavioral difficulties and better social skills in children, particularly among those who come from vulnerable backgrounds.^6^^,^^35^^,^^37^^,^^38^ The quality of centre-based childcare provided in each country may therefore play a role with regard to children's behavioral outcomes.

We found a positive association between children's informal childcare attendance and elevated levels of internalizing symptoms between 7–9 and 10–13 years. This aligns with a study conducted in Britain which found that high use of non-institutionalized care during early childhood was linked to increased levels of teacher-rated anti-social behavior up to the age of seven years.^39^^,^^45^ In Switzerland, it was reported that children who visited a playgroup were more aggressive at ages seven and eight and had more self-reported symptoms of delinquency and substances use at age 13 if they were cared for by acquaintances rather than their parents.^35^ This is consistent with our study, which showed that children who attended informal childcare were more likely to experience externalizing symptoms between the ages of 7–9 and 10–13 years. It may be that informal childcare, provided by persons who are neither properly trained nor supervised to care and stimulate young children, is a poor substitute for parental care and on average does not meet the quality standards of centre-based childcare. Also, children who are cared for by relatives or family friends may not have the same opportunities to interact with children their own age and may therefore have less prosocial skills compared to children attending centre-based childcare.

Our study found no evidence that maternal post-partum depression modifies the relationship between early childcare arrangements and children's internalizing and externalizing symptoms. This result differs from a study conducted in Canada which found that access to high quality childcare buffered the consequences of maternal depression on children's emotional and behavioral problems. One of the differences with our study is that maternal depression was ascertained when the child was in the pre-school age range, whereas we controlled for depression earlier in the child's life.^14^^,^^46^ Another study found that maternal depressive symptoms were most strongly associated with children's increased internalizing and externalizing problems as children grew older.^46^

We also found that low maternal education status appears to positively modify the effect of centre-based and informal childcare on children's internalizing and externalizing symptoms. This aligns with a study which found that children who came from a more favorable socioeconomic background reaped more benefits of childcare compared to children who came from a more disadvantaged background.^7^ This may be because centre-based childcare may not be enough to reverse the negative consequences of coming from a disadvantaged background.^7^ Children coming from more adverse situations are already at a greater disadvantage in terms of their future academic achievement, educational attainment, and cognitive and social development.^47^ This may be because they have a more unstable home environment with fewer stimulating and educational activities, experience less positive parenting practices, and experience more early life stressors compared to children coming from families with a higher socio-economic status.^47^ All of these factors may, therefore, play a stronger role on children's socio-emotional development and may prevent early childcare from having any real impacts on children's development as universal programs may not address the needs of children coming from very diverse backgrounds.^48^ For child's sex, boys had a small increase in externalizing symptoms between 10 and 13 years compared to girls if they attended informal childcare. This interaction may reflect increased externalizing symptoms that boys tend to exhibit compared to girls, who may show more internalizing symptoms, due to various societal expectations and different parenting and caretaking styles given boys and girls are.^49^^,^^50^ Lastly, the stratified results based on the assessment tool used to measure internalizing and externalizing symptoms did not substantially differ from the main results. There was, however, a substantial decrease in heterogeneity in the stratified results which may indicate that the ALSPAC and GENR studies are more similar to each other and the DNBC, ELFE, EDEN, and INMA cohorts, which in turn may be more similar to one another. This could be due to similar study designs or potential country level factors.

Our study has some limitations which need to be acknowledged. First, an important issue in testing the association between childcare attendance and children's later outcomes is the role of confounding characteristics which influence both early education and mental health. Our analyses control for key confounding variables (family socioeconomic characteristics, parental mental health), however we cannot entirely rule out residual confounding, as we limited covariates to variables available in all child cohorts included. Nevertheless, all the covariates we included in this study were derived from a Directed Acyclic Graph (DAG) and no major confounding variable was missing. Second, some children attended different types of childcare over time. Yet we chose to focus on centre-based childcare, as in some participating cohorts, information on childcare attendance was only collected once. This may have introduced some misclassification bias, as studies have shown that children who attend multiple childcare arrangements may display more behavioral problems, possibly in part due to insecure attachment with adults that care for them.^51^ However, this is likely to represent only a minority of children included in our statistical analyses as trends in Europe have found that families who enroll their child into centre-based childcare use centre-based childcare as their primary source of childcare.^52^ Third, parent-reported children's internalizing and externalizing symptoms may underestimate their offspring's psychological difficulties.^53^ However, the use of standardized measures of mental health which have previously been validated is likely to reduce this type of bias.^9^ Fourth, neither the age at which children started early childcare nor if they attend part-time or full-time childcare was measured in our study. These two factors may contribute to children's internalizing and externalizing symptoms, and further research should incorporate these variables. Fifth, the time period of data collection of each cohort may have seen different ECEC policies which may have impacted early childcare attendance. We were unable to adjust for different policy changes within each country, and this limitation must be considered when analyzing the results.

Despite these limitations, our study also has strengths which should be highlighted. First, this is a federated analysis of individual level data from six different parent-child cohort studies across five European countries. The geographic diversity of our study sample provides enhanced location coverage across Europe, improves the generalizability of our results and facilitates intra- and inter-population comparisons. Second, the consistent harmonization of variables across cohort studies reduced between-study heterogeneity and strengthened the generalizability, external validity and reproducibility of findings. Third, we were able to account for key confounding variables and test whether the reported associations varied across family socioeconomic position and child sex, providing additional insight.

### Conclusion

Our study, based on data from six epidemiological cohorts of parents and children recruited across five European settings, highlights complex associations between different types of early childcare arrangements and children's emotional and behavioral outcomes in mid-childhood and early adolescence. Our results indicate that children attending centre-based childcare have lower levels of internalizing symptoms as they grow up, whereas those who attend informal care have higher levels. In children whose mothers have low educational level and in boys these associations appear to be enhanced and the beneficial effects of centre-based childcare are not observed. Nevertheless, overall access to quality centre-based childcare could be a propitious way of promoting child well-being and psychological development in the mid and long-term. In addition, children from socioeconomically disadvantaged families require special attention, as they may not sufficiently benefit from universal ECEC.

## Contributors

KMB, DA and MM conceptualized and designed the present study. Interpretation of the results. KMB, DA, and TC planned and performed the statistical and data analysis. KMB drafted the article. DA, TM, AE, HEM, PWJ, MW, AMNA, KSL, LGS, RSB, FBZ, JJ, MV, BH, MAC, ARG, and MM provided insights on the interpretation of the data and additional analyses to perform. All authors read and revised this manuscript critically for important intellectual content and finally approved this version of the manuscript for submission.

## Data sharing statement

The datasets generated and analyzed during the current study are not publicly available due to data regulations and for ethical reasons, considering that this information might compromise research participants’ consent because our participants only gave their consent for the use of their data by the original team of investigators. However, all the code for the data management and analysis are open sourced and publicly available: https://github.com/katybarry/nonparental-care-attendance-and-childrens-behaviors.git.

## Declaration of interests

The authors have no conflicts of interest to declare that are relevant to the content of this article.

## Appendix A Supplementary data

Supplementary Information revised
