# Supplementary material for: Early childcare arrangements and children's internalizing and externalizing symptoms: an individual participant data meta-analysis of six prospective birth cohorts in Europe
Source: Lancet Reg Health Eur. 2024 Aug 21;45:101036. doi: 10.1016/j.lanepe.2024.101036 (PMC11387227; doi:10.1016/j.lanepe.2024.101036)
Supplement: Supplementary Information revised [file mmc1.docx]

**Supplementary Appendix**

**Ethics approval**

**ALSPAC**

Ethical approval for the study was obtained from the ALSPAC Ethics and Law Committee and the Local Research Ethics Committees. Informed consent for the use of data collected via questionnaires and clinics was obtained from participants following the recommendations of the ALSPAC Ethics and Law Committee at the time Pregnant women resident in Avon, UK with expected dates of delivery 1st April 1991 to 31st December 1992 were invited to take part in the study.

**Generation R**

The general design, all research aims and the specific measurements in the Generation R Study have been approved by the Medical Ethical Committee of Erasmus MC, University Medical Center Rotterdam. Participants need to give written informed consent for each phase of the study (fetal, preschool, childhood and adolescence period).

**DNBC**

The Danish National Birth Cohort (DNBC) is a nationwide longitudinal cohort that enrolled pregnant women through their general practitioners during early gestation (weeks 6 to 12) (Olsen et al., 2001). Written informed consent was obtained from all participants at recruitment, and again during followed up.

**INMA**

The study has been approved by Ethical Committee of each participating centre and written consent was obtained from participating parents. Data for this study comes from INMA-Asturias, INMA-Gipuzkoa, INMA-Sabadell and INMA-Valencia subcohorts.

**EDEN**

The study received approval from the ethics committee (CCPPRB) of Kremlin Bicêtre on 12 December 2002 and from CNIL (Commission Nationale Informatique et Liberté), the French data privacy institution. All subjects gave their informed consent for inclusion before they participated in the study. Consent for the child was obtained from both parents after the child's birth

**ELFE**

Ethical approvals for data collection in maternity units and for each data collection wave during follow-up were obtained from the national advisory committee on information processing in health research (CCTIRS: Comité Consultatif sur le Traitement de l’Information en matière de Recherche dans le domaine de la Santé), the national data protection authority (CNIL: Comission Nationale Informatique et Liberté) and, in case of invasive data collection such as biological sampling, the committee for protection of persons engaged in research (CPP: Comité de Protection des Personnes). The ELFE study was also approved by the national committee for statistical information (CNIS: Conseil National de l’Information Statistique). Informed consent was signed by the parents or the mother alone, with the father being informed of his right to deny consent for participation.

**Consent to participate**

**ALSPAC**

Participants who returned completed questionnaires (either by post or by email) provided written consent to participate.

**Generation R**

Participants provided written informed consent for each phase of the study (fetal, preschool, childhood and adolescence period).

**DNBC**

Written informed consent was obtained from all participants at recruitment, and again during followed up.

**INMA**

Written consent was provided by all participating parents at the beginning of the study.

**EDEN**

Consent for the child was obtained from both parents after the child's birth

**ELFE**

Informed consent was signed by the parents or the mother alone, with the father being informed of his right to deny consent for participation.

Table 1: Percentage missing data on covariates included in the study of associations between childcare attendance and children’s emotional and behavioural difficulties in the EU Child Cohort Network (ALSPAC, GENR, DNBC, INMA, EDEN, ELFE).

| **Variable** | **Missing (in percentage %)** |
| --- | --- |
| Mother’s age | 0·7 |
| Mother’s educational status | 10·9 |
| Mother’s employment status | 4·5 |
| Mother’s post-partum depression | 10·0 |
| Parental separation | 5·6 |
| Child’s sex | 0·01 |
| Only child | 1·3 |
| Child’s gestational week of birth | 0·1 |
| Child’s birth weight in grams | 0·8 |

Table 2: Description of cohort-specific exposure and outcome measures in the study of associations between childcare attendance and children’s emotional and behavioural difficulties in the EU Child Cohort Network (ALSPAC, GENR, DNBC, INMA, EDEN, ELFE)*.

|  | ALSPAC | GENR | DNBC | INMA | EDEN | ELFE |
| --- | --- | --- | --- | --- | --- | --- |
| **Early childcare arrangement** |  |  |  |  |  |  |
| *0 - 1 years* |  | measured | measured | measured | measured | measured |
| *1 - 2 years* | measured | measured | measured | measured | measured | measured |
| *2 - 3 years* | measured | measured |  | measured | measured | measured |
| *3 - 4 years* | measured | measured |  |  | measured |  |
| **Internalising and externalising symptoms (percentiles)** |  |  |  |  |  |  |
| *5 - 6 years* | CBCL | CBCL |  |  | SDQ | SDQ |
| *7 - 9 years* | CBCL | CBCL | SDQ | CBCL | SDQ |  |
| *10 - 13 years* | CBCL | CBCL | SDQ | SDQ |  |  |
| measured | Early childcare attendance: Derived from variables evaluating all types of childcare which the child benefited from prior to school entry. If mothers reported that the child went to a centre-based childcare facility the harmonized variable was coded 1 or 0. The "exactAge" function was used to ensure that variable is based on children’s exact rather than average age at the time of data collection. | | | | | |
| CBCL | Child Behaviour Checklist (CBCL) complete subscale (Internalizing symptoms: anxious-depressed, withdrawn-depressed, somatic complaints), parent-reported  CBCL complete subscale (Externalizing symptoms: rule breaking behaviour, aggressive behaviour), parent-reported | | | | | |
| SDQ | Strengths and Difficulties Questionnaire (SDQ) complete subscale (Internalising symptoms: peer and emotional problems), parent-reported  SDQ complete subscale (Externalising symptoms: hyperactivity/inattention and conduct problems), parent-reported | | | | | |
| Standardisation process for the internalising and externalising symptoms percentile scores | Children’s internalising symptoms (signs of anxiety/depression, withdrawn behaviour and/or somatic complaints) and children’s externalising symptoms (attention difficulties and delinquent and/or aggressive behaviours [1] were reported by parents using either the Strengths and Difficulties Questionnaire (SDQ) [2] or Child Behavioural Checklist (CBCL) [3]. To achieve equivalence between cohorts in instances where different instruments and/or scale items were used, SDQ and CBCL scores were standardized from 0 to 100, with higher scores signifying higher levels of difficulties [26]. If participants did not answer more than 25% of the questions addressing internalising and externalising behaviours, they were labelled as missing. Otherwise, the prorated total scare score was calculated (the total prorated score equals the total raw score based on completed items divided by the number of items which have been responded to multiplied by the total number of items in the scale) [4]. Then, the score was standardized in order to take into account the different scales used to measure internalising and externalising behaviours [4].  1. Nader JL, Harris JR, Cadman T (2021) Report on the life course trajectories leading from early internalizing and externalizing indicators to subsequent psychopathology outcomes in childhood: Lifecycle report D6.1. https://lifecycle-project.eu/our-findings/public-deliverables/. Accessed 3 Aug 2023  2. Goodman R (1997) The Strengths and Difficulties Questionnaire: a research note. J Child Psychol Psychiatry 38:581–586  3. Bilenberg N (1999) The Child Behaviour Checklist (CBCL) and related material: standardisation and validation in Danish population based and clinically based samples. Acta Psychiatr Scand Suppl 398:2–52. <https://doi.org/10.1111/j.1600-0447.1999.tb10703.x>  4. Guides & Manuals. LifeCycle n.d. https://lifecycle-project.eu/for-scientists/guides-manuals/ (accessed January 10, 2022)  ^*ALSPAC: Avon Longitudinal Study of Parents and Children ; GENR: The Generation R study ; DNBC: Danish National Birth Cohort ; INMA: INfancia y Medio Ambiente Project ; EDEN: Étude des Déterminants pré Et postnatals du Développement de la santé de l'enfant ; ELFE: Étude Longitudinale Française depuis l'Enfance ;^ | | | | | |


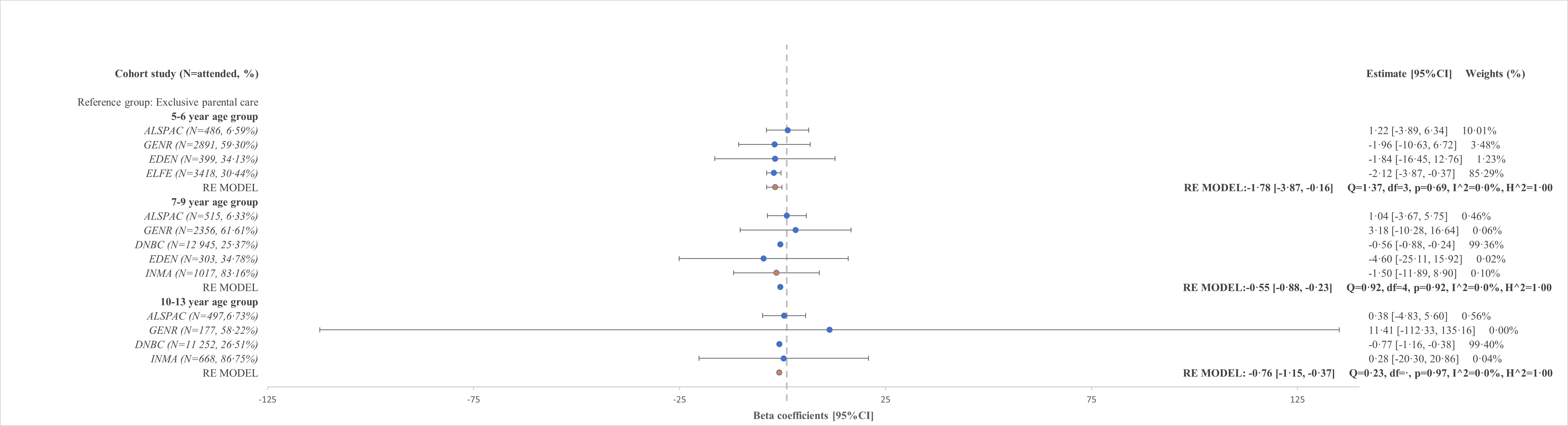
Figure 1: Adjusted associations between any centre-based childcare between ages 0 and 4 years and children’s internalising symptoms between 5-6, 7-9, and 10-13 years in child cohort studies included in the EU Child Cohort Network (ALSPAC, GENR, DNBC, INMA, EDEN, ELFE)*.

^*ALSPAC: Avon Longitudinal Study of Parents and Children ; GENR: The Generation R study ; DNBC: Danish National Birth Cohort ; INMA: INfancia y Medio Ambiente Project ; EDEN: Étude des Déterminants pré Et postnatals du Développement de la santé de l'enfant ; ELFE: Étude Longitudinale Française depuis l'Enfance^

^A two-stage individual participant data (IPD) meta-analysis was performed. The reference group was children who were exclusively cared for by their parents. Linear regression models were performed separately on each cohort for each given age bracket and then cohort-specific coefficients and standard errors were combined using random-effects meta-analysis with a restricted estimate maximum likelihood (REML) approach to attain overall effect estimates. The beta coefficient is provided with the accompanying 95% Confidence Interval (CI) are provided for each cohort and the meta-analysed result with the heterogeneity statistics are also provided.^

^For the ALSPAC, GENR, DNBC, ELFE, and EDEN cohorts, linear regression models were adjusted for maternal age at child’s birth, maternal education at child’s birth, maternal employment status at child’s birth, parental divorce status, mother’s first child, child’s sex, child’s weight at birth, and child’s gestational age at birth. For INMA, model was adjusted for all the previous variables except for maternal post-partum depression and parental divorce status due to unavailability.^

Figure 2: Adjusted associations between any centre-based childcare between ages 0 and 4 years and children’s externalising symptoms between 5-6, 7-9, and 10-13 years in child cohort studies included in the EU Child Cohort Network (ALSPAC, GENR, DNBC, INMA, EDEN, ELFE)*.

^*ALSPAC: Avon Longitudinal Study of Parents and Children ; GENR: The Generation R study ; DNBC: Danish National Birth Cohort ; INMA: INfancia y Medio Ambiente Project ; EDEN: Étude des Déterminants pré Et postnatals du Développement de la santé de l'enfant ; ELFE: Étude Longitudinale Française depuis l'Enfance^
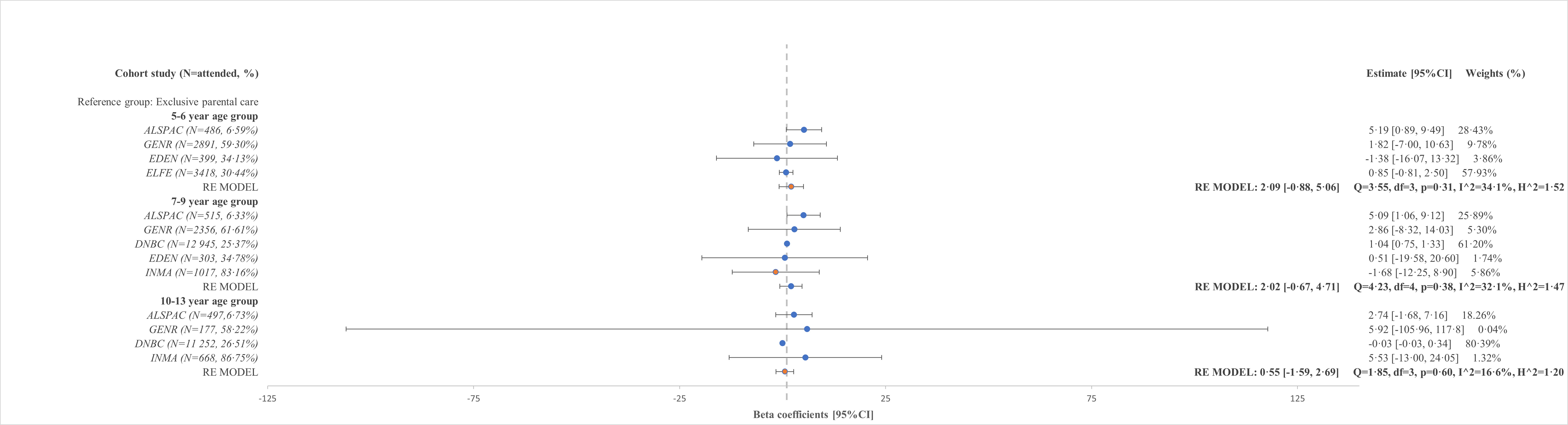


^A two-stage individual participant data (IPD) meta-analysis was performed. The reference group was children who were exclusively cared for by their parents. Linear regression models were performed separately on each cohort for each given age bracket and then cohort-specific coefficients and standard errors were combined using random-effects meta-analysis with a restricted estimate maximum likelihood (REML) approach to attain overall effect estimates^

^The beta coefficient is provided with the accompanying 95% Confidence Interval (CI) are provided for each cohort and the meta-analysed result with the heterogeneity statistics are also provided.^

^For the ALSPAC, GENR, DNBC, ELFE, and EDEN cohorts, linear regression models were adjusted for maternal age at child’s birth, maternal education at child’s birth, maternal employment status at child’s birth, parental divorce status, mother’s first child, child’s sex, child’s weight at birth, and child’s gestational age at birth. For INMA, model was adjusted for all the previous variables except for maternal post-partum depression and parental divorce status due to unavailability.^

Figure 3: Adjusted associations between any informal childcare vs. exclusive parental childcare between ages 0 up to age 4 years and internalising symptoms’ percentile scores between 5-6, 7-9, and 10-13 years in child cohort studies included in the EU Child Cohort Network (ALSPAC, GENR, DNBC, INMA, EDEN, ELFE)*


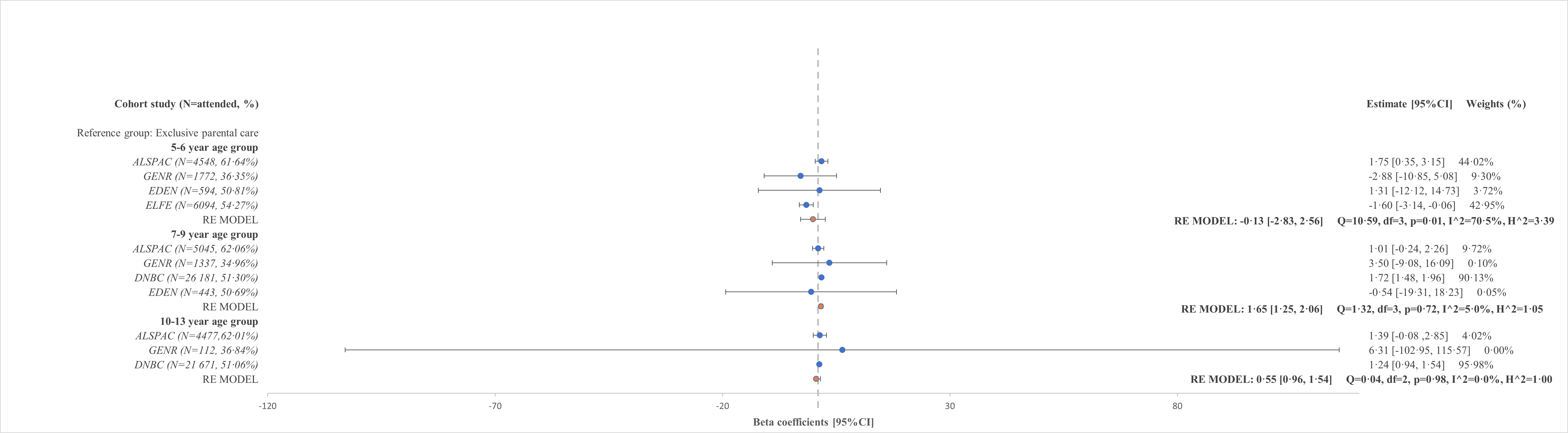


^*ALSPAC: Avon Longitudinal Study of Parents and Children ; GENR: The Generation R study ; DNBC: Danish National Birth Cohort ; INMA: INfancia y Medio Ambiente Project ; EDEN: Étude des Déterminants pré Et postnatals du Développement de la santé de l'enfant ; ELFE: Étude Longitudinale Française depuis l'Enfance^

^A two-stage individual participant data (IPD) meta-analysis was performed. The reference group was children who were exclusively cared for by their parents. Linear regression models were performed separately on each cohort for each given age bracket and then cohort-specific coefficients and standard errors were combined using random-effects meta-analysis with a restricted estimate maximum likelihood (REML) approach to attain overall effect estimates^

^The beta coefficient is provided with the accompanying 95% Confidence Interval (CI) are provided for each cohort and the meta-analysed result with the heterogeneity statistics are also provided.^

^For the ALSPAC, GENR, DNBC, ELFE, and EDEN cohorts, linear regression models were adjusted for maternal age at child’s birth, maternal education at child’s birth, maternal employment status at child’s birth, parental divorce status, mother’s first child, child’s sex, child’s weight at birth, and child’s gestational age at birth. For INMA, model was adjusted for all the previous variables except for maternal post-partum depression and parental divorce status due to unavailability.^

Figure 4: Adjusted associations between any informal childcare between ages 0 and 4 years and children’s externalising symptoms between 5-6, 7-9, and 10-13 years in child cohort studies included in the EU Child Cohort Network (ALSPAC, GENR, DNBC, INMA, EDEN, ELFE)*.


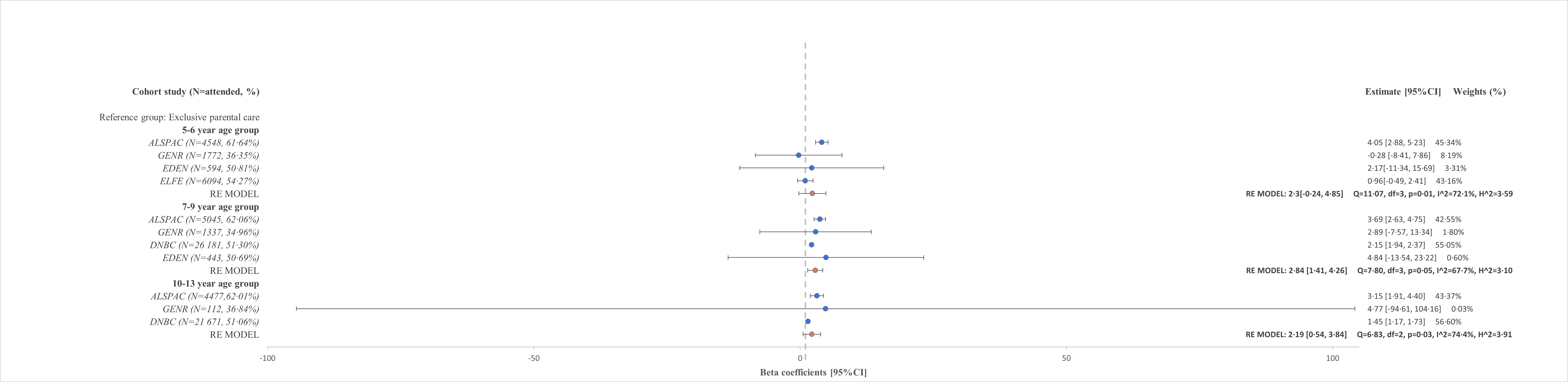


^*ALSPAC: Avon Longitudinal Study of Parents and Children ; GENR: The Generation R study ; DNBC: Danish National Birth Cohort ; INMA: INfancia y Medio Ambiente Project ; EDEN: Étude des Déterminants pré Et postnatals du Développement de la santé de l'enfant ; ELFE: Étude Longitudinale Française depuis l'Enfance^

^A two-stage individual participant data (IPD) meta-analysis was performed. The reference group was children who were exclusively cared for by their parents. Linear regression models were performed separately on each cohort for each given age bracket and then cohort-specific coefficients and standard errors were combined using random-effects meta-analysis with a restricted estimate maximum likelihood (REML) approach to attain overall effect estimates^

^The beta coefficient is provided with the accompanying 95% Confidence Interval (CI) are provided for each cohort and the meta-analysed result with the heterogeneity statistics are also provided.^

^For the ALSPAC, GENR, DNBC, ELFE, and EDEN cohorts, linear regression models were adjusted for maternal age at child’s birth, maternal education at child’s birth, maternal employment status at child’s birth, parental divorce status, mother’s first child, child’s sex, child’s weight at birth, and child’s gestational age at birth. For INMA, model was adjusted for all the previous variables except for maternal post-partum depression and parental divorce status due to unavailability.^


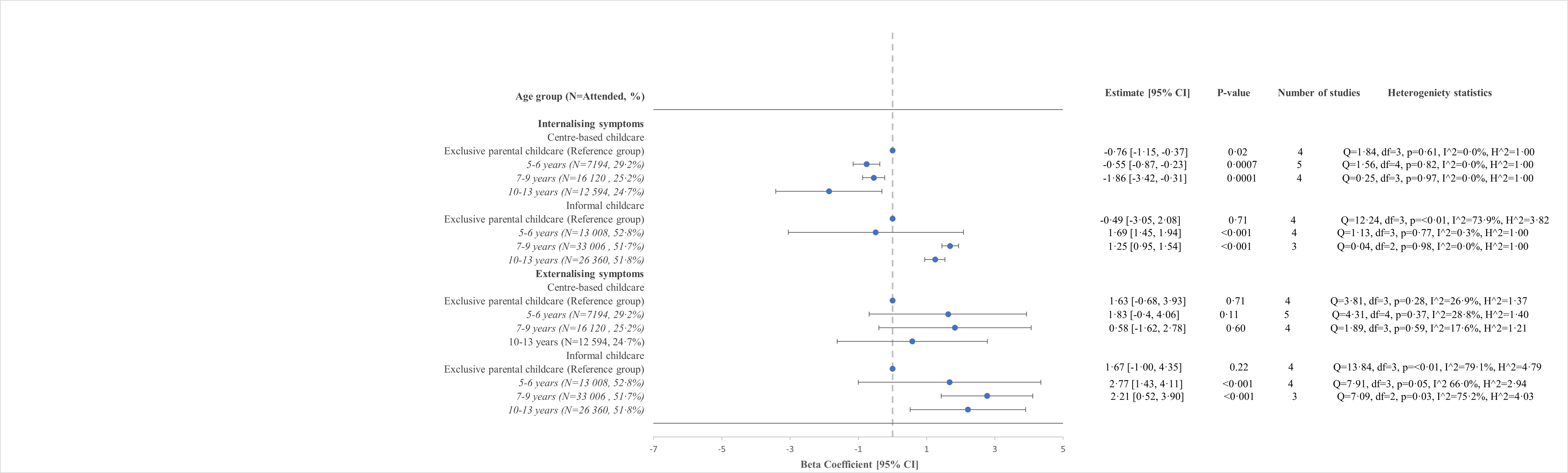
Figure 5: Adjusted associations between any centre-based childcare and informal childcare between ages 0 and 3 years and children’s internalising and externalizing symptoms between 5-6, 7-9, and 10-13 years in child cohort studies included in the EU Child Cohort Network (ALSPAC, GENR, DNBC, INMA, EDEN, ELFE)*.

^*ALSPAC: Avon Longitudinal Study of Parents and Children ; GENR: The Generation R study ; DNBC: Danish National Birth Cohort ; INMA: INfancia y Medio Ambiente Project ; EDEN: Étude des Déterminants pré Et postnatals du Développement de la santé de l'enfant ; ELFE: Étude Longitudinale Française depuis l'Enfance^

^A two-stage individual participant data (IPD) meta-analysis was performed. The reference group was children who were exclusively cared for by their parents. Linear regression models were performed separately on each cohort for each given age bracket and then cohort-specific coefficients and standard errors were combined using random-effects meta-analysis with a restricted estimate maximum likelihood (REML) approach to attain overall effect estimates^

^The beta coefficient is provided with the accompanying 95% Confidence Interval (CI) are provided for each cohort and the meta-analysed result with the heterogeneity statistics are also provided.^

^For the ALSPAC, GENR, DNBC, ELFE, and EDEN cohorts, linear regression models were adjusted for maternal age at child’s birth, maternal education at child’s birth, maternal employment status at child’s birth, parental divorce status, mother’s first child, child’s sex, child’s weight at birth, and child’s gestational age at birth. For INMA, model was adjusted for all the previous variables except for maternal post-partum depression and parental divorce status due to unavailability.^

Table 3. Early childhood education and care information for each cohort participating in the EU Child Cohort Network based on the data collection time period.

| Cohort (country) | Recruitment period for cohorts | Early childhood education and care structure |
| --- | --- | --- |
| ALSPAC (England) | 1991-1992 | The central government was not directly involved in childcare provision, and local government authorities as well as non-governmental bodies mainly provided childcare [1,2]. There was also no guarantee for a place in childcare for children prior to primary school. The cost of childcare was expensive, with limited aid for low-income families. The Children Act 1989 established the regulatory framework to ensure childcare providers met certain health, safety, and welfare standard though not until 2008 did the Early Years Foundation Stage (EYFS) framework lay out specific staff qualifications and child-to-staff ratios [3]. |
| GENR (The Netherlands) | 2002-2006 | The central government provided grants to local authorities in order to manage childcare services and the cost of childcare was divided between parents, employers, and the government [4]. There was also no guarantee for a place in childcare for children prior to primary school. A regulatory framework in was established which required childcare providers to meet certain health, safety, and educational quality. Further information on the quality of early childcare has been provided elsewhere [5]. |
| DNBC (Denmark) | 1995-2002 | In 1999, around 92% of all children aged 3-5 years and more than 50% of children aged 0-2 years attended public childcare in 1999 [6]. The cost of childcare was relatively cheap compared to other countries as parents were only allowed to pay 30% of the childcare costs [6]. At this time, children were not guaranteed a spot in childcare. The public childcare system was universal and primary based on children’s needs [6]. Public childcare was highly subsidized and offered to children prior to primary school entry [6]. Standards and regulations on the health, safety, educational curriculum, and staff-to-child were implemented by the central government in which each childcare provider had to abide by [7]. |
| INMA (Spain) | 1997-2008 | Public funding of early childcare was aimed at making childcare services accessible to families [8]. The Spanish Education law created a framework for the quality of early childhood education and required both public and private childcare services to meet certain educational and staff care standards (specifically for childcare after the age of 3 years) [8]. Children were guaranteed a spot in childcare after the age of 3 years, but no guarantee was provided before the age of 3 years, though local authorities were encouraged to offer enough space for early childcare. The public childcare services offered affordable costs but limited placement for children under the age of 3 years [8]. |
| EDEN (France)  ELFE (France) | 2003-2006  2011 | Childcare at this time, and currently, is highly centralized, with the government setting the rules and regulations around costs of childcare and regulations that childcare services must abide by [9]. During this time period, the government continued to expand availability in publicly subsidized centre-based childcares for children, created assistance programs for low-income families to afford childcare, provided grant to childcare facilities, and enforced mandatory trainings for professional staff to ensure quality childcare [10]. One way the government has dealt with increasing demand of childcare is introducing more subsidies for childminders, who currently handle the majority of childcare in France to this day. There was not a guaranteed place for children in childcare before primary school [9]. Continuing from this, France made some policy changes after the financial crisis in 2008 in which more benefits were provided to low-income and one-parent families seeking childcare services [11] |

^*ALSPAC: Avon Longitudinal Study of Parents and Children ; GENR: The Generation R study ; DNBC: Danish National Birth Cohort ; INMA: INfancia y Medio Ambiente Project ; EDEN: Étude des Déterminants pré Et postnatals du Développement de la santé de l'enfant ; ELFE: Étude Longitudinale Française depuis l'Enfance^

[1] Lewis J. Continuity and Change in English Childcare Policy 1960–20001. Soc Polit Int Stud Gend State Soc 2013;20:358–86. https://doi.org/10.1093/sp/jxt013.

[2] Faulkner D, Coates EA. Early childhood policy and practice in England: twenty years of change. Int J Early Years Educ 2013;21:244–63. https://doi.org/10.1080/09669760.2013.832945.

[3] Participation E. Children Act 1989 n.d. https://www.legislation.gov.uk/ukpga/1989/41/contents (accessed July 5, 2024).

[4] McKenney S, Letschert J, Kloprogge J. Early childhood education in the Netherlands: the first steps. Educ. 4 8 Years Olds Re-Des. Sch. Entr. Phase, CIDREE/DVO; 2007, p. 53–62.

[5] Helmerhorst K, Riksen-Walraven M, Gevers Deynoot-Schaub M, Tavecchio L, Fukkink R. Child Care Quality in The Netherlands Over the Years: A Closer Look. Early Educ Dev 2014;26. https://doi.org/10.1080/10409289.2014.948784.

[6] The Impact of Family-Friendly Policies in Denmark and Sweden on Mothers’ Career Interruptions Due to Childbirth n.d. https://www.iza.org/publications/dp/1050/the-impact-of-family-friendly-policies-in-denmark-and-sweden-on-mothers-career-interruptions-due-to-childbirth (accessed July 5, 2024).

[7] de la Porte C, Larsen TP, Lundqvist Å. Still a poster child for social investment? Changing regulatory dynamics of early childhood education and care in Denmark and Sweden. Regul Gov 2023;17:628–43. https://doi.org/10.1111/rego.12492.

[8] Ramírez M. Education and Childcare Policies. An Overview of the Spanish Case. Ric Pedagog E Didattica 2010;5. https://doi.org/10.6092/issn.1970-2221/1774.

[9] Fagnani J. Recent reforms in childcare and family policies in France and Germany: What was at stake? Child Youth Serv Rev 2012;34:509–16. https://doi.org/10.1016/j.childyouth.2011.10.011.

[10] L’accueil des jeunes enfants en France : etat des lieux et pistes d’amel | vie-publique.fr 2024. https://www.vie-publique.fr/rapport/25923-laccueil-des-jeunes-enfants-en-france-etat-des-lieux-et-pistes-damel (accessed July 5, 2024).

[11] Thévenon O, Adema W, Ali N. Family policy in France and Europe: Recent changes and effects of the crisis. Popul Soc 2014;512:1–4.

Table 4. Pooled interaction estimates between a) mother’s post-partum depression, b) mother’s education level, and c) the child’s sex and childcare attendance with regard to children’s internalising and externalising symptoms in the EU Child Cohort Network (β coefficient, accompanying p-value)

| **Internalising symptoms** |  |  |  |
| --- | --- | --- | --- |
| ***Age group*** | **Interaction term** | **B coefficient** | **P value** |
| *5-6 years* | Centre-based childcare*mother’s post-partum depression (yes) | -3·85 | 0·66 |
| *7-9 years* | Centre-based childcare*mother’s post-partum depression (yes) | 0·32 | 0·82 |
| *10-13 years* | Centre-based childcare*mother’s post-partum depression (yes) | -0·52 | 0·75 |
| *5-6 years* | Informal childcare*mother’s post-partum depression (yes) | -2·08 | 0·74 |
| *7-9 years* | Informal childcare*mother’s post-partum depression (yes) | -1·07 | 0·28 |
| *10-13 years* | Informal childcare*mother’s post-partum depression (yes) | 0·05 | 0·96 |
| *5-6 years* | Centre-based childcare* mother’s education level (yes) | 0·12 | 0·93 |
| *7-9 years* | Centre-based childcare*mother’s education level (yes) | 12·58 | 0·22 |
| *10-13 years* | Centre-based childcare* mother’s education level (yes) | 1·27 | <0·001 |
| *5-6 years* | Informal childcare* mother’s education level (yes) | 49·74 | <0·001 |
| *7-9 years* | Informal childcare* mother’s education level (yes) | 44·22 | <0·001 |
| *10-13 years* | Informal childcare* mother’s education level (yes) | 44·86 | <0·001 |
| *5-6 years* | Centre-based childcare*child’s sex (female) | 2·4 | 0·43 |
| *7-9 years* | Centre-based childcare* child’s sex (female) | -1·28 | 0·05 |
| *10-13 years* | Centre-based childcare* child’s sex (female) | -0·09 | 0·91 |
| *5-6 years* | Informal childcare* child’s sex (female) | 1·15 | 0·54 |
| *7-9 years* | Informal childcare* child’s sex (female) | 0·08 | 0·93 |
| *10-13 years* | Informal childcare* child’s sex (female) | 0·56 | 0·35 |
| **Externalising symptoms** |  |  |  |
| ***Age group*** | **Interaction term** | **B coefficient** | **P value** |
| *5-6 years* | Centre-based childcare*mother’s post-partum depression (yes) | -2·54 | 0·76 |
| *7-9 years* | Centre-based childcare*mother’s post-partum depression (yes) | 0·1 | 0·93 |
| *10-13 years* | Centre-based childcare*mother’s post-partum depression (yes) | -0·76 | 0·61 |
| *5-6 years* | Informal childcare*mother’s post-partum depression (yes) | -0·82 | 0·88 |
| *7-9 years* | Informal childcare*mother’s post-partum depression (yes) | 0·08 | 0·93 |
| *10-13 years* | Informal childcare*mother’s post-partum depression (yes) | -0·39 | 0·75 |
| *5-6 years* | Centre-based childcare* mother’s education level (yes) | 2·68 | 0·03 |
| *7-9 years* | Centre-based childcare* mother’s education level (yes) | 12·84 | 0·19 |
| *10-13 years* | Centre-based childcare* mother’s education level (yes) | 2·22 | 0·01 |
| *5-6 years* | Informal childcare * mother’s education level (yes) | 49·73 | <0·0001 |
| *7-9 years* | Informal childcare * mother’s education level (yes) | 47·34 | <0·0001 |
| *10-13 years* | Informal childcare * mother’s education level (yes) | 46·62 | <0·0001 |
| *5-6 years* | Centre-based childcare*child’s sex (female) | -1·95 | 0·48 |
| *7-9 years* | Centre-based childcare* child’s sex (female) | -0·81 | 0·15 |
| *10-13 years* | Centre-based childcare* child’s sex (female) | -0·89 | 0·22 |
| *5-6 years* | Informal childcare* child’s sex (female) | 1·21 | 0·47 |
| *7-9 years* | Informal childcare* child’s sex (female) | 0·41 | 0·83 |
| *10-13 years* | Informal childcare* child’s sex (female) | -1·21 | 0·03 |
| Interaction variables were coded as such: Mother’s low education status (any education at or below upper secondary, post-secondary or non-tertiary school as classified by the International Standard Classification of Education 97/2011 (ISCED-97/2011) (yes/no), mother experienced post-partum depression within one year of child’s birth (yes/no), child’s sex (male/female) | | | |

Table 5: Adjusted associations between childcare attendance between ages 0 up to age 4 years and children’s internalizing and externalizing symptoms by age group, stratified based on the type of measurement tool used to assess children’s internalizing and externalizing symptoms in the EU Child Cohort Network.

|  | **Cohorts in analysis** | **Estimate, 95%CI** | **P value** | **Heterogeneity statistics** |
| --- | --- | --- | --- | --- |
| **Cohorts using the SDQ to assess children’s levels of internalizing and externalizing symptoms** | | | | |
| **Internalizing symptoms** |  |  |  |  |
| Centre-based childcare |  |  |  |  |
| *5-6 years 3817 (30·70%)* | EDEN, ELFE | -2·12 [-3·85,-0·38] | 0·02 | Q=0·00, df=1, p=0·97, I^2^=0·0%, H^2^=1·00 |
| *7-9 years 13 249 (23·10%)* | DNBC, EDEN | -0·56 [-0·88, -0·24] | 0·001 | Q=0·15, df=2, p=0·70, I^2^=0·0%, H^2^=1·00 |
| *10-13 years 11 920 (19·70%)* | DNBC, INMA | -0·77 [-1·16,-0·38] | 0·0001 | Q=0·01, df=1, p=0·92, I^2^=0·0%, H^2^=1·00 |
| Informal childcare |  |  |  |  |
| *5-6 years 6688 (53·8%)* | EDEN, ELFE | -1·56 [-3·09, -0·03] | 0·045 | Q=0·18, df=1, p=0·67, I^2^=0·0%, H^2^=1·00 |
| *7-9 years 26 624 (43·20%)* | DNBC, EDEN | 1·72 [1·48,1·96] | <0·0001 | Q=0·06, df=1, p=0·81, I^2^=0·0%, H^2^=1·00 |
| *10-13 years 21 671 (35·90%)* | DNBC | 1·22 [0·46, 1·98] | <0·0001 | Only DNBC cohort |
| **Externalizing symptoms** |  |  |  |  |
| Centre-based childcare |  |  |  |  |
| *5-6 years 3817 (30·70%)* | EDEN, ELFE | 0·82 [-0·82,2·46] | 0·33 | Q=0·09, df=1, p=0·77, I^2^=0·0%, H^2^=1·00 |
| *7-9 years 13 249 (23·10%)* | DNBC, EDEN | 1·04 [0·75,1·33] | <0·0001 | Q=0·00, df=1, p=0·96, I^2^=0·0%, H^2^=1·00 |
| *10-13 years 11 920 (19·70%)* | DNBC, INMA | -0·03 [-0·40,0·34] | 0·87 | Q=0·35, df=1, p=0·56, I^2^=0·0%, H^2^=1·00 |
| Informal childcare |  |  |  |  |
| *5-6 years 6688 (53·8%)* | EDEN, ELFE | 0·97 [-0·47, 2·42] | 0·19 | Q=0·03, df=1, p=0·86, I^2^=0·0%, H^2^=1·00 |
| *7-9 years 26 624 (43·20%)* | DNBC, EDEN | 2·16 [1·94,2·37] | <0·0001 | Q=0·08, df=1, p=0·77, I^2^=0·0%, H^2^=1·00 |
| *10-13 years 21 671 (35·90%)* | DNBC | 1·41 [0·68,2·15] | <0·0001 | Only DNBC cohort included |
| **Cohorts using the CBCL to assess children’s levels of internalizing and externalizing symptoms** | | | | |
| **Internalizing symptoms** |  |  |  |  |
| Centre-based childcare |  |  |  |  |
| *5-6 years 3377 (23·50%)* | ALSPAC, GENR, INMA | 0·40 [-4·00,4·81] | 0·86 | Q=0·38, df=1, p=0·54, I^2^=0·0%, H^2^=1·00 |
| *7-9 years 3888 (24.80)* | ALSPAC, GENR, INMA | 0·85 [-3·24, 4·93] | 0·68 | Q=0·32, df=2, p=0·85, I^2^=0·0%, H^2^=1·00 |
| *10-13 years 674 (4·70%)* | ALSPAC, GENR | 0·40 [-4·80,5·61] | 0·88 | Q=0·03, df=1, p=0·86, I^2^=0·0%, H^2^=1·00 |
| Informal childcare |  |  |  |  |
| *5-6 years 6320 (44·00%)* | ALSPAC, GENR | 1·16 [-1·88,4·20] | 0·45 | Q=1·26, df=1, p=0·26, I^2^=20·9%, H^2^=1·26 |
| *7-9 years 6382 (44·50%)* | ALSPAC, GENR | 1·04 [-0·21,2·28] | 0·10 | Q=0·15, df=1, p=0·70, I^2^=0·0%, H^2^=1·00 |
| *10-13 years 4689 (32·70%)* | ALSPAC, GENR | 1·39 [-0·08,2·85] | 0·10 | Q=0·01, df=1, p=0·93, I^2^=0·0%, H^2^=1·00 |
| **Externalizing symptoms** |  |  |  |  |
| Centre-based childcare |  |  |  |  |
| *5-6 years 3377 (23·50%)* | ALSPAC, GENR, INMA | 4·54 [0·67,8·41] | 0·02 | Q=0·45, df=1, p=0·50, I^2^=0·0%, H^2^=1·00 |
| *7-9 years 6382 (40·80%)* | ALSPAC, GENR, INMA | 4·09 [0·53,7·66] | 0·02 | Q=1·43, df=2, p=0·49, I^2^=0·0%, H^2^=1·00 |
| *10-13 years 674 (4·70%)* | ALSPAC, GENR | 0·40 [-4·80, 5·61] | 0·22 | Q=0·03, df=1, p=0·86, I^2^=0·0%, H^2^=1·00 |
| Informal childcare |  |  |  |  |
| *5-6 years 6320 (44·00%)* | ALSPAC, GENR | 3·84 [1·98, 5·69] | <0·0001 | Q=1·07, df=1, p=0·30, I^2^=6·2%, H^2^=1·07 |
| *7-9 years 6382 (44·50%)* | ALSPAC, GENR | 3·68 [2·62, 4·74] | <0·0001 | Q=0·02, df=1, p=0·88, I^2^=0·0%, H^2^=1·00 |
| *10-13 years 4689 (32·70%)* | ALSPAC, GENR | 3·15 [1·91,4·40] | <0·0001 | Q=0·00, df=1, p=0·97, I^2^=0·0%, H^2^=1·00 |

^ALSPAC: Avon Longitudinal Study of Parents and Children ; GENR: The Generation R study ; DNBC: Danish National Birth Cohort ; INMA: INfancia y Medio Ambiente Project ; EDEN: Étude des Déterminants pré Et postnatals du Développement de la santé de l'enfant ; ELFE: Étude Longitudinale Française depuis l'Enfance ; Strengths and Difficulties Questionnaire (SDQ), Child Behaviour Checklist (CBCL)^

^The beta coefficient is provided with the accompanying 95% Confidence Interval (CI) are provided for each cohort and the meta-analysed result with the heterogeneity statistics are also provided.^

^For the ALSPAC, GENR, DNBC, ELFE, and EDEN cohorts, linear regression models were adjusted for maternal age at child’s birth, maternal education at child’s birth, maternal employment status at child’s birth, parental divorce status, mother’s first child, child’s sex, child’s weight at birth, and child’s gestational age at birth. For INMA, model was adjusted for all the previous variables except for maternal post-partum depression and parental divorce status due to unavailability.^
